# APOBEC3 as a driver of genetic intratumor heterogeneity

**DOI:** 10.1080/23723556.2021.2014734

**Published:** 2022-01-03

**Authors:** Subramanian Venkatesan, Mihaela Angelova, Jirina Bartkova, Samuel F. Bakhoum, Jiri Bartek, Nnennaya Kanu, Charles Swanton

**Affiliations:** a Cancer Evolution and Genome Instability Laboratory, The Francis Crick Institute, London, UK; b Cancer Research UK Lung Cancer Centre of Excellence, UCL Cancer Institute, University College London, London, UK; c Genome Integrity Unit, Danish Cancer Society Research Center, Copenhagen, Denmark; d Division of Genome Biology, Department of Medical Biochemistry and Biophysics, Science for Life Laboratory, Karolinska Institute, Stockholm, Sweden; e Human Oncology and Pathogenesis Program, Memorial Sloan Kettering Cancer Center, New York, New York, USA; f Department of Radiation Oncology, Memorial Sloan Kettering Cancer Center, New York, New York, USA; g Department of Medical Oncology, University College London Hospitals NHS Foundation Trust, London, UK

**Keywords:** APOBEC, non-small cell lung cancer, breast cancer, intratumor heterogeneity, chromosomal instability

## Abstract

Our recent study revealed that APOBEC3B is upregulated during the preinvasive stages of non-small cell lung cancer and breast cancer. In addition to its role in mediating single nucleotide variants, we propose that APOBEC3 promotes copy number intratumor heterogeneity prior to invasion, providing a substrate for cancer evolution.

Analysis of sequencing data from tumor samples has revealed extensive intratumor heterogeneity at the single nucleotide as well as copy number level. Genomic intratumor heterogeneity is clinically relevant since it has been linked to immune evasion, anti-cancer treatment resistance and disease progression. Therefore, identifying drivers of intratumor heterogeneity could provide targets to intercept treatment resistance, disease progression and potentially even cancer initiation in high-risk patients.

Mutational signature analysis of sequencing data has revealed molecular underpinnings of mutagenic processes in cancer. One of the most pervasive signatures among different cancer types is an APOBEC3-mediated mutational signature. Apolipoprotein B mRNA-editing enzyme, catalytic polypeptide-like 3 (*APOBEC3; A3*) is a family of cytidine deaminases consisting of 7 genes which are a part of the innate immune system. APOBEC3 forms a barrier against viral and transposon replication through cytidine deaminase-dependent and independent mechanisms. APOBEC3-mediated mutagenesis in viral, transposon and genomic DNA occurs through deamination of cytidines present in single-stranded DNA into uracils. Uracil can either be reverted back into a cytosine by the DNA damage repair system or be replaced by a thymine or guanine by the DNA replication machinery creating a single nucleotide mutation. This APOBEC3 mutational signature consists of C-to-T and C-to-G mutations in TCA and TCT trinucleotide motifs. The APOBEC3 family members A3A and A3B appear to be the main drivers of this signature, with the former appearing to be a more dominant mutator.^1^ Our group and others have shown that APOBEC3 is a major driver of subclonal mutagenesis and cell-to-cell variation within several cancer types.^2^

## APOBEC3 activity occurs in preinvasive lesions and cancer

In a recent study,^3^ we investigated *APOBEC3* expression in non-small cell lung cancer (NSCLC) development and found extensive nuclear immunohistochemical staining for A3A and A3B in preinvasive lesions of lung adenocarcinoma and lung squamous cell carcinoma, but absent in normal epithelial cells. We confirmed these observations in independent gene expression datasets of NSCLC, revealing that preinvasive samples with moderate dysplasia had transcriptional upregulation of different *APOBEC3* genes, including *A3A* and *A3B*, relative to normal epithelium. Besides NSCLC, we confirmed an increase in *A3B* gene expression in preinvasive breast lesions. In addition, whole exome sequencing of matched preinvasive and invasive lesions in 2 NSCLC patients revealed that APOBEC3-mediated subclonal mutagenesis can occur at the preinvasive stage prior to malignant transformation.

## A3B induces chromosomal instability before malignant transformation

We identified that *A3B*-expressing cells exhibited a slower DNA replication fork speed relative to isogenic *A3B* knockout cells, supporting data from other groups,^4^ collectively suggesting that A3B contributes to replication stress. Isogenic *A3B* wild type and *A3B* knockout cell lines were studied throughout different cell cycle phases. *A3B* wild type cells contained more Fanconi anemia complementation group D2 (FANCD2) foci in prometaphase, more metaphase breaks at the fragile histidine triad diadenosine triphosphatase (*FHIT*) common fragile site locus, more FANCD2-flanked ultrafine bridges and more tumor protein 53-binding protein 1 (TP53BP1, best known as 53BP1) nuclear bodies in the G1 cell cycle phase relative to *A3B* knockout cells. These observations suggest that A3B-mediated replication stress promotes double-stranded DNA breaks at late replicating common fragile sites. Interestingly, these phenotypes manifested only upon the addition of low-dose aphidicolin or the expression of a RAS oncogene, suggesting A3B might need some level of replication stress in order to induce genome instability. *A3B* wild type cells displayed higher frequencies of chromosome missegregation relative to *A3B* knockout cells. Furthermore, within an *A3B*-inducible mouse model of lung cancer, *A3B*-overexpressing cells contained higher levels of phosphorylated replication protein A (RPA) and higher frequencies of chromosome missegregation relative to *A3B*-deficient cells, indicative of replication stress and chromosomal instability, respectively. Besides cytidine deamination on ssDNA, recent studies have also implicated APOBEC3 in chromatin modification.^5^ It is therefore conceivable that APOBEC3 can promote genome instability through distinct but not mutually exclusive mechanisms. Depending on the cell line and cancer type, other APOBEC3 family members such as A3A have recently also been shown to reduce DNA replication fork speed and to be involved in promoting chromosomal instability.^6,7^

## APOBEC3 as a therapeutic target in cancer

**Figure 1.**
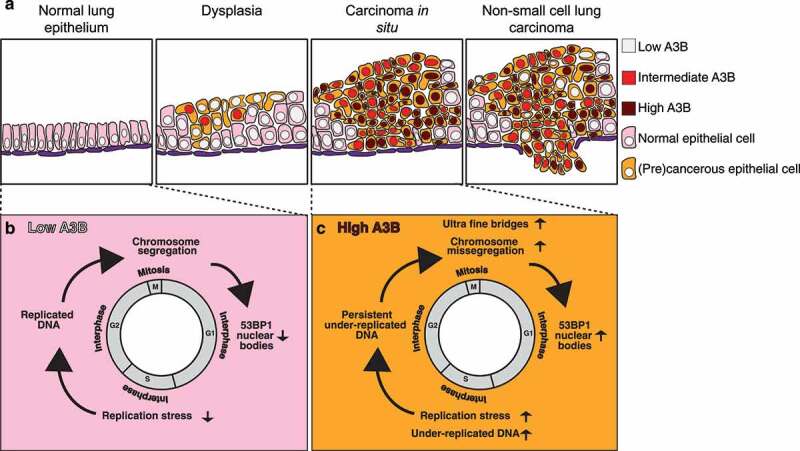
Upregulation of A3B levels in preinvasive cancer promotes genome instability.

Taken together, these findings are of interest since in NSCLC, chromosomal instability in the context of somatic DNA copy number heterogeneity has been associated with an increased risk of recurrence or death (see Figure 1).^8^ With APOBEC3 family members being drivers of single nucleotide variants and chromosomal instability, it might be attractive to inhibit relevant APOBEC3 family members. Combination therapy with APOBEC3 inhibitors might be able to impede tumor evolution and the acquisition of treatment-resistant clones. A more controversial approach might seek to hyperactivate relevant APOBEC3 family members, beyond an optimal range in which genome instability increases cancer cell fitness, to drive cell autonomous lethality due to excessive genome instability. Recent evidence suggests that *A3B* overexpression and ensuing mutagenesis increases sensitivity to immunotherapies,^9^ although there are also concerns that subclonal neoantigen heterogeneity may fuel an immune suppressive microenvironment and immune evasion.^10^ The timing of APOBEC3 induction and the ensuing clonal or subclonal repertoire of neoantigens may determine sensitization to immune-mediated predation. In summary, our experimental and clinical data suggest that A3B fuels chromosomal instability in the context of somatic copy number diversity in addition to single nucleotide heterogeneity in early NSCLC development, enabling downstream selection and cancer evolution. In the future it will be of interest to address whether eradicating genomically unstable preinvasive cancer cells, or inhibiting APOBEC3 in carcinoma *in situ*, can prevent progression into invasive cancer.

**A**, Apolipoprotein B mRNA-editing enzyme, catalytic polypeptide-like 3 *(APOBEC3; A3)* gene expression increases during early non-small cell lung cancer (NSCLC) evolution. A3B protein levels are especially high in carcinoma *in situ* samples relative to normal lung epithelium. **B** and **C**, example of cells that have low and high A3B levels, respectively. A3B promotes replication stress and the accumulation of under-replicated DNA, leading to the formation of ultrafine bridges, chromosome missegregation and tumor protein 53-binding protein 1 (TP53BP1, best known as 53BP1) nuclear bodies.
